# Activation Mobilizes the Cholesterol in the Late Endosomes-Lysosomes of Niemann Pick Type C Cells

**DOI:** 10.1371/journal.pone.0030051

**Published:** 2012-01-20

**Authors:** Yvonne Lange, Jin Ye, Theodore L. Steck

**Affiliations:** 1 Department of Pathology, Rush University Medical Center, Chicago, Illinois, United States of America; 2 Department of Biochemistry and Molecular Biology, University of Chicago, Chicago, Illinois, United States of America; Consejo Superior de Investigaciones Cientificas, Spain

## Abstract

A variety of intercalating amphipaths increase the chemical activity of plasma membrane cholesterol. To test whether intracellular cholesterol can be similarly activated, we examined NPC1 and NPC2 fibroblasts, since they accumulate large amounts of cholesterol in their late endosomes and lysosomes (LE/L). We gauged the mobility of intracellular sterol from its appearance at the surface of the intact cells, as determined by its susceptibility to cholesterol oxidase and its isotope exchange with extracellular 2-(hydroxypropyl)-β-cyclodextrin-cholesterol. The entire cytoplasmic cholesterol pool in these cells was mobile, exchanging with the plasma membrane with an apparent half-time of ∼3–4 hours, ∼4–5 times slower than that for wild type human fibroblasts (half-time ∼0.75 hours). The mobility of the intracellular cholesterol was increased by the membrane-intercalating amphipaths chlorpromazine and 1-octanol. Chlorpromazine also promoted the net transfer of LE/L cholesterol to serum and cyclodextrin. Surprisingly, the mobility of LE/L cholesterol was greatly stimulated by treating intact NPC cells with glutaraldehyde or formaldehyde. Similar effects were seen with wild type fibroblasts in which the LE/L cholesterol pool had been expanded using U18666A. We also showed that the cholesterol in the intracellular membranes of fixed wild-type fibroblasts was mobile; it was rapidly oxidized by cholesterol oxidase and was rapidly replenished by exogenous sterol. We conclude that a) the cholesterol in NPC cells can exit the LE/L (and the extensive membranous inclusions therein) over a few hours; b) this mobility is stimulated by the activation of the cholesterol with intercalating amphipaths; c) intracellular cholesterol is even more mobile in fixed cells; and d) amphipaths that activate cholesterol might be useful in treating NPC disease.

## Introduction

This study concerns the mobility of intracellular cholesterol and, in particular, its exit from late endosomal and lysosomal (LE/L) compartments. The unesterified cholesterol in the cytoplasm of most cells is scant. We have therefore examined Niemann-Pick C (NPC) cells, because they bear large amounts of cholesterol (along with certain membrane phospholipids) in multilamellar inclusions within their LE/L [1], [2]. This phenotype results from the lack of functional NPC1 and/or NPC2 proteins. NPC2 is a water-soluble cholesterol-binding protein located in the lumen of the LE/L where it appears to shuttle sterol molecules from the membranous inclusions therein to the NPC1 protein in the boundary membranes [3]–[6]. NPC1 is a membrane-spanning, cholesterol-binding protein that facilitates the exit of cholesterol from the boundary membranes of the LE/L by an unknown mechanism [3], [4], [7], [8].

The phenotype of NPC disease strongly suggests that an NPC1/NPC2 pathway provides the major route by which cholesterol normally exits from the LE/L, and it is widely held that in NPC1^−/−^ cells “no known pathway exists for rapidly moving sequestered lysosomal sterol to the plasma membrane” [9]. However, there is evidence that the surfeit of LE/L cholesterol in NPC cells is not trapped there but, rather, turns over on a time scale of hours [10]–[12]. These findings suggest the existence of a second, normally minor route by which cholesterol can leave the LE/L. This unknown pathway is probably critical for prolonging NPC cell survival, so that the therapeutic enhancement of this alternate route might help to alleviate the intracellular accumulation of cholesterol in NPC disease. In fact, two recently reported strategies have described such interventions. In one approach, ingestion of the sterol-binding agent, HPCD, reduced the intracellular cholesterol in cultured NPC1 cells as well as in afflicted animals [8], [9], [13]–[17]. The other strategy was to increase the level of acid sphingomyelinase in the LE/L of NPC1 cells; this too mobilized the sequestered cholesterol by hydrolyzing sphingomyelin, its high-affinity binding partner [18]. The exit pathway for the mobilized LE/L cholesterol in these cases is unknown.

The present study sought to test the hypothesis that activators of plasma membrane cholesterol also mobilize cytoplasmic cholesterol. Active membrane cholesterol is that fraction not held in complexes with polar lipids. The cholesterol in such lipid complexes has a relatively low chemical activity, escape tendency or fugacity [19], [20]. In contrast, active cholesterol appears to have an increased frequency and/or extent of projection into the aqueous environment. In particular, the fraction of plasma membrane cholesterol that exceeds the complexing capacity of its polar lipid partners is more accessible to water-soluble probes such as cholesterol oxidase and β-cyclodextrins. Furthermore, excess plasma membrane cholesterol redistributes down its activity gradient to the endoplasmic reticulum and mitochondria where it elicits homeostatic responses [20], [21].

Membrane-intercalating amphipaths competitively displace sterols from their complexes with phospholipids and thereby activate them [19], [22]–[25]. We have now tested two such amphipaths, 1-octanol and CPZ (chlorpromazine), for their ability to mobilize the large pool of LE/L cholesterol in NPC1 and NPC2 cells. We found that CPZ promoted the net transfer of this cholesterol to serum lipoproteins and that CPZ and octanol accelerated LE/L cholesterol transfer to the plasma membrane. Glutaraldehyde also has been observed to increase the activity of membrane cholesterol [26]–[28]; we now show that it too mobilizes the LE/L cholesterol in NPC1 cells. The surprising nature of some of these results was explored in various control experiments; these indicated that the observed mobilization of intracellular cholesterol was not due to the disruption of cell integrity or permeability barrier but rather to activation of the sterol.

## Results

### Effect of amphipaths on the net transfer of NPC cell cholesterol to serum

**Figure 1:**
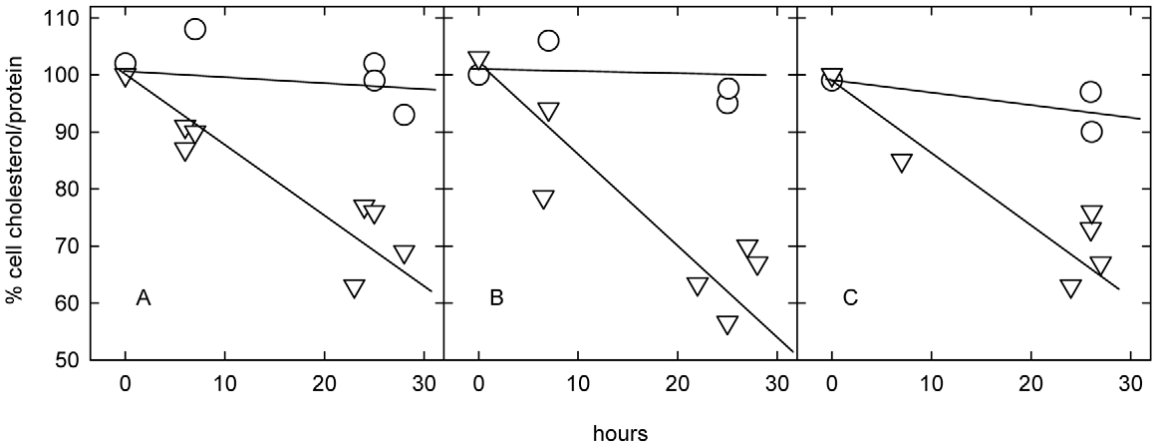
Effect of chlorpromazine on cholesterol transfer from NPC cells to serum. Cells in 6-well plates were grown to confluence in DME10 (*i.e.,* DME containing 10% fetal calf serum). Wells were then incubated at 37°C with 1 ml DME10 without (○) or with 20 µM chlorpromazine (▿). At the indicated times, duplicate pairs of wells were assayed for unesterified cell cholesterol and protein and the data averaged; the remaining wells were given fresh medium and the incubation continued. Panel A, NPC1 line 93.59. Panel B, NPC1 line 93.41. Panel C, NPC2 line 99.04. Data for each cell line were pooled from 2 or 3 experiments.

Figure 1 shows that, as would be expected, the unesterified cholesterol in untreated NPC cells did not change appreciably during an overnight incubation with 10% fetal calf serum. In contrast, the presence of CPZ caused a 30–60% decrease in unesterified cholesterol in the two NPC1 and one NPC2 cell lines. Parallel experiments showed that, at the end of the overnight incubation, 21% of the total cholesterol in NPC1 line 93.59 was in the ester form, compared to 12% in the untreated control. This augmented esterification is in keeping with other evidence that CPZ activates membrane cholesterol [22], [25]; this increases the cholesterol pool in the endoplasmic reticulum and thereby stimulates cholesterol esterification [23], [24]. We infer that the bulk of the cholesterol lost from the cells had been transferred to the serum acceptor; however, such increments could not be measured accurately because of the high cholesterol content of the serum lipoprotein sink.

The subcellular distribution of the unesterified cholesterol remaining after the overnight incubation of NPC cells with serum was estimated by analyzing the biphasic kinetics of the oxidation of this cholesterol at the outer surface of cells after their fixation by glutaraldehyde. (This technique, termed Method B, is fully characterized below.) We see in Table 1 that CPZ stimulated far more loss of cell surface and intracellular cholesterol than can be accounted for by the increment in the esterified fraction mentioned above. We infer that the missing cholesterol had been transferred to the serum. That CPZ drove the redistribution of plasma membrane and intracellular cholesterol to serum implies that it raised the chemical activity of the sterol in the cells more than that in the serum lipoproteins. We also found that the addition of HPCD further enhanced the loss of cholesterol from both the plasma membrane and the intracellular compartments of the CPZ-treated cells (Table 1).

**Table 1:** Effect of chlorpromazine on cholesterol exit from NPC fibroblasts to serum.

Cells	Line	% Serum	CPZ	HPCD	cells PM IC
					µg cholesterol/mg cell protein		
NPC1	93.59	20	−		82.7	36.7	46.0
			+		60.4	25.7	34.7
					(0.73)	(0.70)	(0.75)
	93.59	10	−		75.3	25.5	59.3
			+		59.8	24.0	34.6
					(0.79)	(1.0)	(0.66)
	93.59	10	−		96.9	35.6	61.4
			+	+	53.2	21.5	31.7
					(0.55)	(0.60)	(0.52)
	93.59	10	−		122.8	44.1	78.9
			+	+	68.2	24.5	43.5
					(0.56)	(0.55)	(0.55)
NPC1	93.41	20	−		117.2	28.4	88.8
			+		86.0	25.1	60.9
					(0.73)	(0.88)	(0.68)
	93.41	10	−		92.9	33.6	59.3
			+		74.1	29.0	45.0
					(0.80)	(0.86)	(0.76)
NPC2	99.04	10	−		95.7	36.4	59.3
			+		78.8	23.7	55.1
					(0.82)	(0.65)	(0.93)
	99.04	10	−		87.8	43.6	44.2
			+		59.2	26.1	33.1
					(0.67)	(0.60)	(0.75)

In multiple replicate experiments, duplicate 75 cm^2^ flasks were incubated at 37°C with 10 ml DME containing 10 or 20% fetal calf serum with or without 20 µM chlorpromazine and 0.15% (i.e., ∼1 mM) HPCD. The medium was replenished after 3, 7 and 21 h of incubation as depicted in Figure 1. After 24 h, the distribution of cholesterol between the plasma membrane (PM) and intracellular (IC) pools was determined by fixing the cells and analyzing the time course of their reaction with cholesterol oxidase as described for Method B in Materials and Methods. The parenthetic values represent fractional changes; i.e., quotients of (plus CPZ)/(minus CPZ).

### Exchange of [^3^H]cholesterol between untreated fibroblasts and extracellular cholesterol

**Figure 2:**
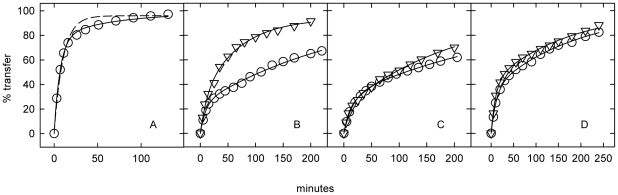
Effect of chlorpromazine on cell [^3^H]cholesterol exchange with cholesterol-cyclodextrin: Method A. These representative experiments were carried out and analyzed as described in Materials and Methods. Panel A, wild-type fibroblasts. Data were fit to a first-order expression (dashed line, R^2^ = 0.975, t_1/2_ = 7 min) as well as to a second-order expression (solid line, R^2^ = 0.998). The latter fit suggests that 21% of the cholesterol was intracellular and that the values for the half-times of the fast and slow processes were 5 min and 44 min. Panel B, NPC1 cell line 93.59. (○), Minus CPZ: 61% of the cholesterol was intracellular and the t_1/2_ values were 7 min and 288 min. (▿), Plus 50 µM CPZ: 71% of the cholesterol was intracellular and the t_1/2_ values were 8 and 47 min. Panel C, NPC1 line 93.41. (○), Minus CPZ: 71% of the cholesterol was intracellular and the t_1/2_ values were 11 and 222 min. (▿), Plus 50 µM CPZ: 79% of the cholesterol was intracellular and the t_1/2_ values were 6 and 138 min. Panel D, NPC2 line 99.04. (○), Minus CPZ: 64% of the cholesterol was intracellular and the t_1/2_ values were 8 and 117 min. (▿), Plus 50 µM CPZ. 59% of the cholesterol was intracellular and the t_1/2_ values were 7 and 115 min.

We tested whether the stimulation of the transfer of intracellular cholesterol to serum by CPZ resulted from cholesterol activation by analyzing the kinetics of [^3^H]cholesterol exchange between uniformly-labeled cells and an “infinite” sink of unlabeled extracellular cholesterol held in HPCD complexes (Method A). First, we examined wild-type fibroblasts (Figure 2A). We found that all of the cell [^3^H]cholesterol became accessible at the cell surface over time; hence, the intracellular fraction must have been entirely mobile. Approximately 80% of the cell [^3^H]cholesterol could be assigned to the fast (plasma membrane) compartment (Table 2), a fraction similar to that reported previously [11]. The half-time for the exchange of the slow (intracellular) compartment was ∼0.75 h, a value concordant with diverse estimates of intracellular cholesterol dynamics [20].

**Table 2:** Dynamics of intracellular [^3^H]cholesterol in wild-type and NPC cells.

Cell type	Line	Treatment	Plasma membrane cholesterol	Intracellular cholesterol	Half-time, min	
			µg/mg cell protein		Fast step	Slow step
Wild-type		None	23.7±3	6.5±0.6	5.3±0.5	47±2
NPC1	93.59	None	31.5±7	70.0±9	6.9±0.4	237±51
		0.8 mM Octanol			4.9±0.8	85±15
		0.05 mM CPZ			3.7±2	41±2
NPC1	93.41	None	25.0±3	75.0±3	6.5±0.5	162±24
		0.8 mM Octanol			10.0±5	129±30
		0.05 mM CPZ			5.0±1	130±8
NPC2	99.04	None	39.1±6	61.0±6	8.4±1	157±27
		0.05 mM CPZ			6.3±1	119±21

The distribution and kinetics of exchange of [^3^H]cholesterol from monolayers of fibroblasts to an HPCD-cholesterol sink were determined and analyzed as described for Method A in Materials and Methods and illustrated in Figure 2. Stated values are means ± average deviations for 2 experiments except for the untreated line 99.04 for which a SD for 5 determinations is given; the average of the nine average deviations was ±15% as was the one standard deviation determined.

Three NPC cell lines were also examined using Method A (Figure 2B–D). Cell cholesterol mass remained nearly constant during these exchange incubations, suggesting chemical equilibrium, while >60% of the [^3^H]cholesterol was transferred to (*i.e.*, exchanged with) the acceptor. Extrapolations of the best fits of the data to infinite time suggested that essentially all of the NPC1 and NPC2 [^3^H]cholesterol exchanged with the extracellular sink in such experiments. Thus, no permanently sequestered pool of intracellular cholesterol was evident. The cholesterol content of the plasma membrane of the NPC cells was roughly comparable to that of wild-type fibroblasts (Table 2). The half-time for its exit to the acceptor was 5–10 min, similar to that found for normal cells in these experiments (Table 2). In contrast, the t_½_ for the exchange of the slow (intracellular) cholesterol compartment in the NPC cells was ∼2.5–4 h, several times slower than the wild-type. These values are similar to those previously reported for NPC1 cells [10], [11]. The cholesterol content of the slowly-exchanging cholesterol pools in the three NPC cell lines was about ten times greater than in the wild-type, so that the intracellular pools amounted to two-thirds or more of the total cell cholesterol. These findings are consistent with numerous earlier studies demonstrating that the excess cholesterol in NPC fibroblasts resides in their LE/L; see, for example, [1], [11], [14], [29], [30]. We therefore take the slowly exchanging pool in the NPC cells to represent their LE/L cholesterol.

### Effect of amphipaths on the exchange of [^3^H]cholesterol between fibroblasts and extracellular cholesterol

Treating NPC1 fibroblasts with either of the two amphipaths accelerated the slow exchange step in Method A (Figure 2B–D and Table 2). The half-times of the slow process in line 93.59 were reduced about 3- and 6-fold by octanol and CPZ, respectively, so that they then resembled the values for the wild-type. For NPC1 line 93.41 and NPC2 line 99.04, the half-time for the slow step was decreased by the two agents by about 25%.

### Kinetics of cholesterol exchange in glutaraldehyde-treated NPC fibroblasts

**Figure 3:**
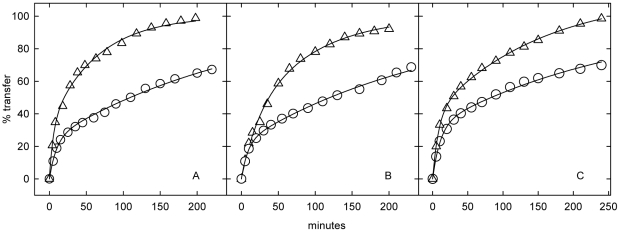
Effect of glutaraldehyde on NPC fibroblast cholesterol dynamics: Method A. Representative experiments showing the exchange of [^3^H]cholesterol between homogeneously-labeled glutaraldehyde-fixed cells and HPCD-cholesterol, as described in Materials and Methods. Panel A, NPC1 cell line 93.59. (○), Unfixed cells: 76% of the cholesterol was intracellular and the t_1/2_ values were 6 and 186 min for the fast and slow compartments. (Δ), Fixed cells: 69% of the cholesterol was intracellular and the t_1/2_ values were 3.7 and 44 min. Panel B, NPC1 cell line 93.41. (○), Unfixed cells: 78% of the cholesterol was intracellular and the t_1/2_ values were 6 and 186 min. (Δ), Fixed cells: 69% of the cholesterol was intracellular and the t_1/2_ values were 15 and 59 min. Panel C, NPC2 cell line 99.04. (○), Unfixed cells: 68% of the cholesterol was intracellular and the t_1/2_ values were 7.5 and 187 min. (Δ), Fixed cells: 74% of the cholesterol was intracellular and the t_1/2_ values were 6 and 105 min.

While glutaraldehyde has been shown to activate membrane cholesterol [26]–[28], we were nevertheless surprised to find that virtually all of intracellular [^3^H]cholesterol in the three lines of NPC cells exchanged with the extracellular HPCD-cholesterol sink after fixation with glutaraldehyde (Figure 3 and Table 3). Furthermore, glutaraldehyde fixation accelerated the slow step (*i.e.,* the movement of LE/L cholesterol to the cell surface) by 3- or 4-fold in the NPC1 cells and by ∼1.7-fold in the NPC2 cells. Glutaraldehyde did not significantly stimulate the already rapid rate of exchange of the plasma membrane [^3^H]cholesterol pool. Treating glutaraldehyde-fixed NPC1 or NPC2 fibroblasts with either of the two intercalating amphipaths, octanol and CPZ, caused little further acceleration of the already rapid exchange of the LE/L cholesterol (Table 3).

**Table 3:** Effect of glutaraldehyde on [^3^H]cholesterol dynamics in fixed fibroblasts.

Cells	Line	Amphipath	Fixation	% intracellular	Half-time, min		
					Fast step	Slow step	n
Wild-type	*	-	−	21.5±1	5.3±0.5	47±2	3
NPC1	93.59*	-	−	70±9	6.9±0.4	237±51	2
		-	+	61±7	7.9±4	53±9	2
		0.8 mM Octanol	+	81±9	4.4±0.3	46±5	2
		0.05 mM CPZ	+	66±5	5.5±0.2	35±4	2
NPC1	93.41*	-	−	75±3.0	6.5±0.5	162±24	2
		-	+	68.5±0.5	9.0±5.0	52±7	2
		0.8 mM Octanol	+	85.5±4.5	3.4±0.6	51.5±0.5	2
		0.05 mM CPZ	+	78±7	3.7±2	41±2	2
NPC2	99.04*	-	−	61±6	8.4±1	157±27	5
		-	+	64±1	6.3±0.9	119±21	3

Cells were pre-equilibrated with [^3^H]cholesterol, fixed or not while still in culture flasks and the exchange of label with an HPCD-cholesterol sink determined and analyzed as described for Method A in Materials and Methods and illustrated in Figure 3. Values for duplicate experiments are means ± average deviations; for n>2, values are means ± S.D.

*Data for untreated control cells were taken from Table 2.

### Kinetics of the oxidation of cholesterol in fixed NPC fibroblasts

**Figure 4:**
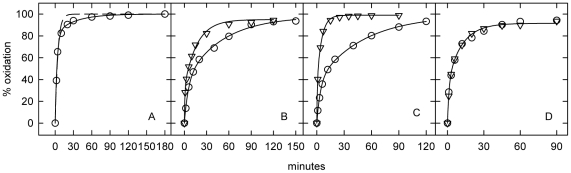
Effect of amphipaths on cholesterol dynamics in fixed fibroblasts: Method B. Representative experiments depicting the action of cholesterol oxidase on glutaraldehyde-treated cells as described in Materials and Methods. Panel A, wild-type fibroblasts. Fits were to a single (dashed line) and a two-exponential expression (solid line). In the two-exponential fit, 18% of cholesterol was intracellular and the t_1/2_ values were 2 and 21 min for the fast and slow compartments. Panel B, effect of CPZ on NPC1 cell line 93.59. (○), Minus CPZ: 61% of the cholesterol was intracellular and the t_1/2_ values were 3.6 and 35 min. (Δ), Plus 50 µM CPZ: 65% of the cholesterol was intracellular and the t_1/2_ values were 0.5 and 12 min. Panel C, effect of octanol on NPC1 cell line 93.41. (○), Minus octanol: 61% of the cholesterol was intracellular and the t_1/2_ values were 2.7 and 35 min. (Δ), Plus 0.8 mM octanol: 64% of the cholesterol was intracellular and the t_1/2_ values were 0.4 and 3.5 min. Panel D, effect of CPZ on NPC2 cell line 99.04. (○), Minus CPZ and (Δ) plus 50 µM CPZ were both fitted by the same parameters: 60% of the cholesterol was intracellular and the t_1/2_ values were 1 and 8 min.

A second measure of the mobility of intracellular cholesterol is the rate of its oxidation by cholesterol oxidase at the cell surface (Method B). Figure 4A shows that the time-course of cholesterol oxidase attack on the cholesterol mass in fixed wild-type fibroblasts was biphasic and that the entire cellular pool was accessible at the cell surface, just as in the case of [^3^H]cholesterol exchange with extracellular HPCD-cholesterol in unfixed cells (Figure 2A). Both the fast and slow processes varied in proportion to the concentration of cholesterol oxidase (not shown). 82% of the cholesterol in the wild-type fibroblasts was attributable to the plasma membrane (Table 4), a value similar to that found for unfixed fibroblasts (Figure 2 and Table 2) and in an earlier study [11].

**Table 4:** Effect of amphipaths on cholesterol dynamics in fixed fibroblasts.

Cells	Line	Treatment	% intracellular	Half-time, min		
				Fast step	Slow step	n
Wild-type		None	18±3	2.2±0.7	23±4	3
NPC1	93.59	None	63±11	2.0±1	24±10	6
		Octanol	67±6	0.20±0.03	5.0±0.4	2
		CPZ	60±2	0.4±0.1	9.3±2	2
NPC1	93.41	None	59±3	3.0±1	40±8	4
		Octanol	63±14	0.49±0.08	5.6±2.5	4
NPC2	99.04	None	56±2	0.95±0.05	12±2	2
		CPZ	58±1	1.0±0.2	10±0.5	2

Cells were suspended, fixed, washed and preincubated for ∼5 min at 37°C with 0.05 mM CPZ or 0.8 mM octanol. Cholesterol oxidase was then added to initiate time courses which were analyzed as described in Method B of Materials and Methods and illustrated in Figure 4. Values are means ± average deviations for 2 independent experiments or means ± SD for n>2.

Fixed NPC1 and NPC2 fibroblasts also gave biphasic time courses of oxidation (Figure 4B–D). The half-times of oxidation of the slow pools in the fixed NPC cells (Table 4) were generally similar to those obtained for [^3^H]cholesterol exchange (Table 3) and significantly greater than those seen in unfixed cells (Table 2). The addition of either CPZ or octanol to the fixed cells further mobilized the slow compartment in the two NPC1 lines but not in NPC2 cells.

Increasing the glutaraldehyde concentration from 1% to 4% and raising the temperature of fixation of the NPC1 cells from 0°C to 37°C further enhanced both the fast and slow rate processes in subsequent tests (not shown). The effects of the glutaraldehyde treatments were mimicked by 4% formaldehyde; however, 100 mM acetaldehyde had no effect on the oxidation of cell cholesterol by cholesterol oxidase (not shown).

### Net transfer of cholesterol from fixed cells to extracellular HPCD

**Figure 5:**
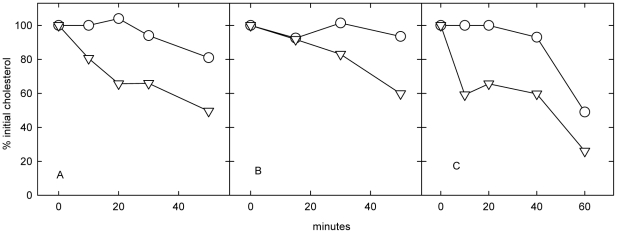
Effect of chlorpromazine on net cholesterol transfer from fixed NPC cells to cyclodextrin. Cells were dissociated, washed, incubated with 1% glutaraldehyde for 30 min on ice, washed again and resuspended in PBS. Aliquots of 100 µl (containing∼100 µg protein) were placed in tubes and made to 0 (O) or 40 µM chlorpromazine (∇). After a 10 min pre-incubation at 37°C, HPCD was added to a concentration of 5% (∼36 mM) to each tube and the incubation continued. At the times indicated, pairs of tubes were chilled, the cells washed and unesterified cholesterol determined. The input (not exposed to CPZ or HPCD) defined the initial cholesterol. Panel A, NPC line 93.59. Panel B, NPC1 line 93.41. Panel C, NPC2 line 99.04. Shown are representatives of several similar experiments.

Figure 5 shows that the initial rate of transfer of cholesterol mass from glutaraldehyde-fixed cells to extracellular cyclodextrin was significantly stimulated by CPZ in all three cell lines. In particular, about 50, 40 and 70% of the total cell cholesterol disappeared from the three NPC cell lines during a 1-hour incubation. (Fixation inactivates cholesterol esterification, ruling out this activity as a cause of the loss of cell cholesterol. Furthermore, cholesterol depletion was dependent on the presence of HPCD.) From Table 1, we know that no more than one-third of the cholesterol in these NPC cells resides in the plasma membrane; also, the preponderance of the intracellular cholesterol is in the LE/L [1], [11], [14], [29], [30]. Since, presumably, not all of the plasma membrane cholesterol in the CPZ-treated cells was removed during the incubation, a large fraction of the cholesterol transferred to the HPCD must have come from the LE/L.

### Visualizing cholesterol mobility in fibroblasts fixed with formaldehyde

**Figure 6:**
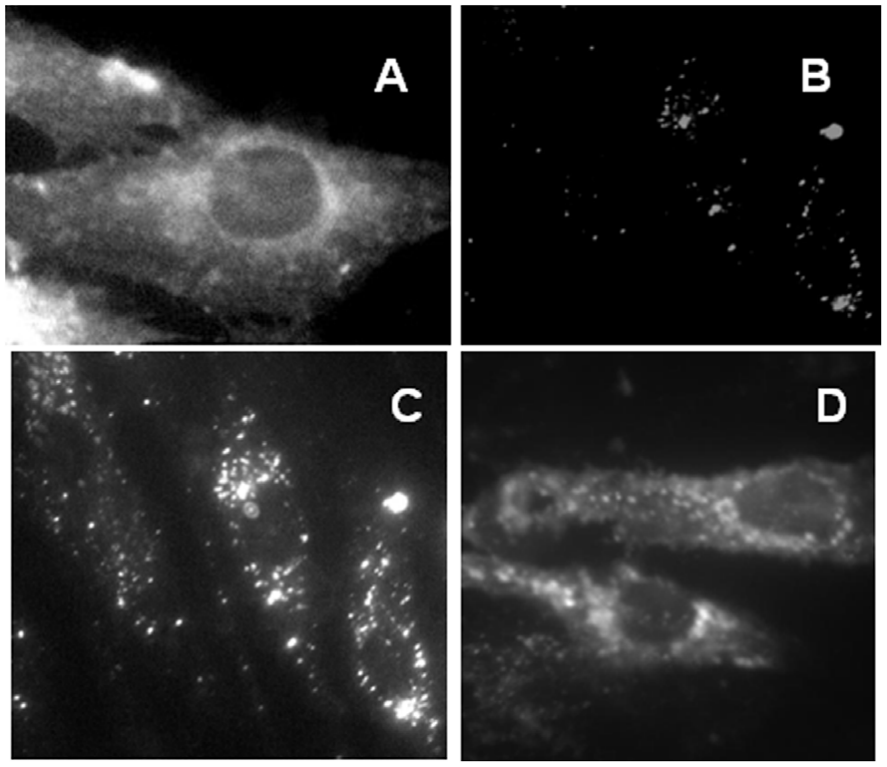
Dynamics of intracellular cholesterol visualized by filipin staining. Wild-type fibroblasts were grown on Falcon culture slides, rinsed with PBS (pH 7.4), fixed with 4% formaldehyde in PBS for 30 min on ice, rinsed and incubated with or without cholesterol oxidase. Staining with filipin III (10 µg/ml PBS) was at room temperature for 30 min in the dark. Images were obtained using an Olympus IX81 microscope and analyzed with Image J software. There was no detectable fluorescence in the absence of filipin. Panel A, formaldehyde-fixed control fibroblasts. Panel B, as in Panel A, except that the cells were treated after fixation with 0.6 U/ml cholesterol oxidase for 10 min at 37°C. Panel C, same field as Panel B but shown at a high intensity setting. Panel D, cells were fixed and treated with cholesterol oxidase as in Panel B, then washed and replenished by incubation in PBS containing 1.1 mg/ml cyclodextrin bearing 30 µg/ml cholesterol in PBS for 10 min at 37°C, then rinsed before staining.

To independently confirm the high mobility of intracellular cholesterol in fixed cells, we used the cholesterol-specific fluorescent probe, filipin, to follow cholesterol dynamics after formaldehyde treatment. (Glutaraldehyde gave a high autofluorescence background in such experiments; fortunately, as mentioned earlier, formaldehyde also mobilized cholesterol.) As widely observed, filipin weakly stained the plasma membrane as well as numerous round cytoplasmic vesicles in the fixed wild-type fibroblasts (Figure 6A). As is typical of LE/L in these cells, the stained vesicles clustered near the nuclei and sometimes capped them irregularly; *e.g*., see [30], [31]. A 10 min incubation of these fibroblasts with cholesterol oxidase abolished nearly all of the filipin staining (Figure 6B). That the cholesterol oxidase treatment did not perturb the cytoarchitecture but simply removed most of the sterol is made clear by Figure 6C; here, viewing the same field as in panel B at high image intensity revealed that normal cell morphology was preserved.

For Figure 6D, cells that had been fixed and then cleared with cholesterol oxidase as in panel B were replenished by incubating them for 10 min with HPCD-cholesterol. The replenished cells regained the normal staining pattern of the plasma membrane and intracellular vesicles, except that they were generally brighter than untreated controls. We infer that cholesterol can move within minutes and in both directions between the cell surface and well-preserved intracellular membrane compartments in fixed wild-type fibroblasts as well as in NPC cells.

### Control experiments

**Figure 7:**
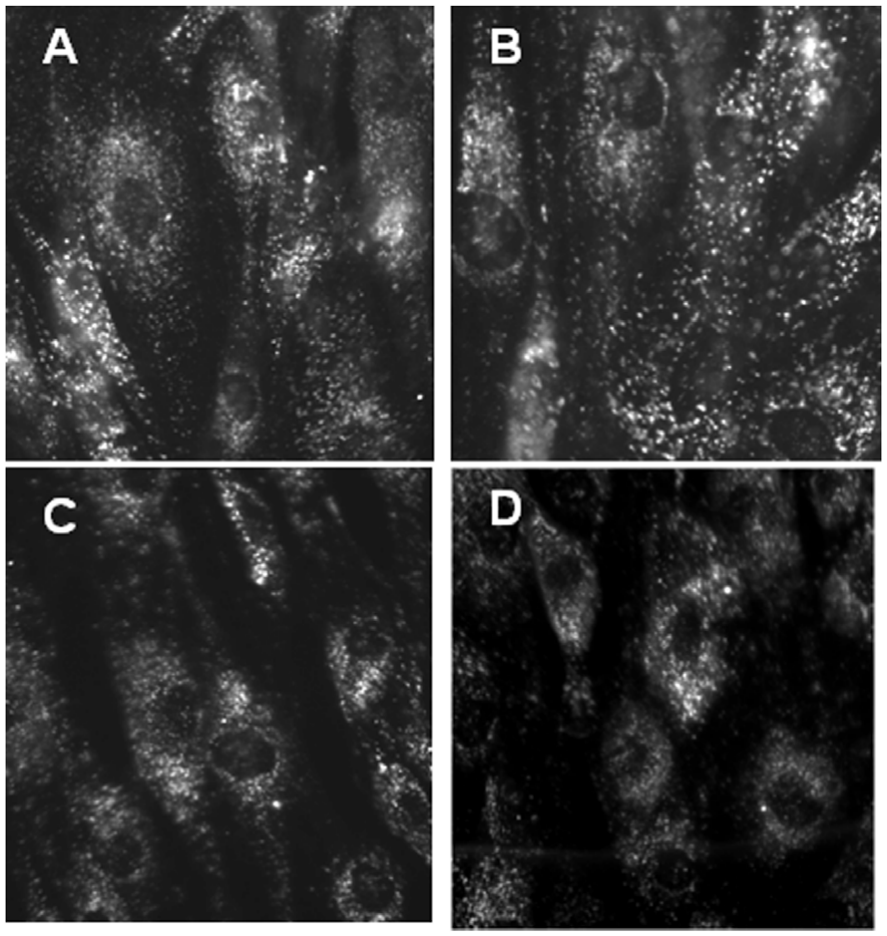
Morphological integrity of cells treated with chlorpromazine. Wild-type (panels A and B) and NPC1 line 93.59 cells (panels C and D) growing on cover slips in wells containing DME10 were fed FITC-Dextran (3 mg/ml) for 3 h at 37°C. 20 µM chlorpromazine was then added to two wells (panels B and D) and all were incubated for an additional hour at 37°C. The cells were fixed on ice for 30 min with 1% glutaraldehyde in PBS (pH 8); autoflorescence was quenched with 20 mM glycine in PBS for 5 min at 37°C and the cells rinsed and examined in a Zeiss Axiovert 100 fluorescence microscope.

Could the increase in intracellular cholesterol accessibility caused by amphipaths have reflected cell disruption? Countering this hypothesis, we note that cholesterol mass was not lost from the NPC cells during the exit of most of the cellular [^3^H]cholesterol to cyclodextrin. Furthermore, we observed that NPC fibroblasts incubated with amphipaths and the cyclodextrin-cholesterol exchange partner had a normal appearance by phase contrast microscopy. Similarly, CPZ had no visible effect on the number, size or intensity of staining of the fluorescent endocytic compartments in unfixed wild-type and NPC1 fibroblasts that had previously ingested fluorescently-labeled dextran (Figure 7). We also found that the effect of CPZ on the mobilization of LE/L cholesterol was mostly reversed by washing out the agent prior to assay (not shown). While cyclodextrin has been shown to promote LE/L sterol excretion by causing lysosome exocytosis [32], we observed only a small release of β-galactosidase, an LE/L acid hydrolase, from the NPC fibroblasts – and this was not increased by octanol or CPZ (not shown). Finally, while cyclodextrin can promote LE/L sterol excretion following its ingestion and transfer to the LE/L [15], the rapid exit of intracellular [^3^H]cholesterol to extracellular cyclodextrin was not blocked (but rather stimulated) in glutaraldehyde-fixed cells in which the endocytosis of HPCD should not occur.

**Figure 8:**
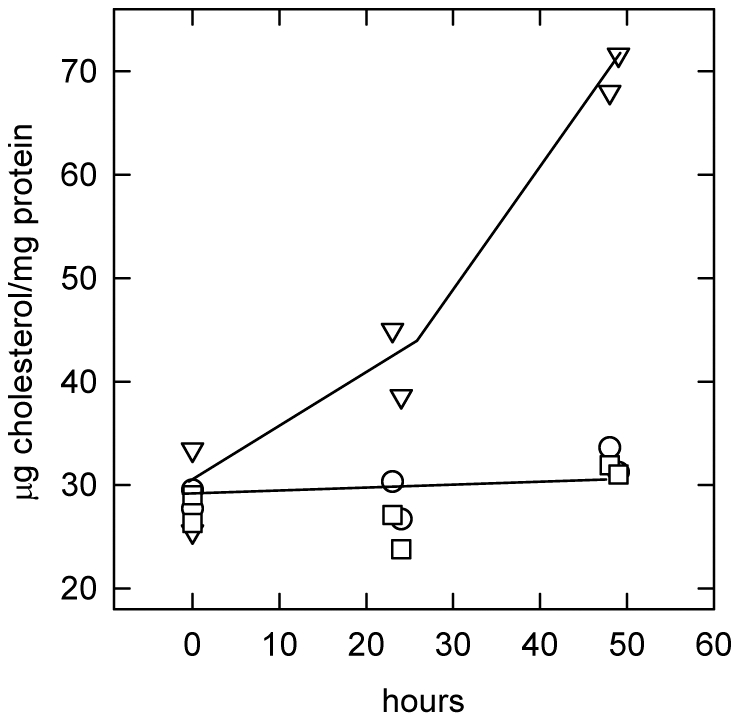
Effect of chlorpromazine and U18666A on cell cholesterol accumulation. Replicate flasks of wild-type fibroblasts were incubated in DME10 containing no additions (○), 20 µM CPZ (□) or 5 µM U18666A (▿). At the times indicated, cells were dissociated with trypsin, washed and assayed in duplicate for unesterified cholesterol and protein. Data from two experiments were pooled.

It was also relevant to assess whether CPZ might have caused cholesterol to accumulate in the LE/L. CPZ is one of many amphipathic amines that induce phospholipidosis; that is, the accumulation of large amounts of polar (glyco- and phospho-) lipids in the LE/L of wild-type cells [33], [34]. Some agents in this category also appear to cause cholesterol to build up in the LE/L; for example, see [35]. However, we could not find strong evidence in the literature that CPZ actually causes an increase in intracellular cholesterol. In fact, upon examination, we found that CPZ did not alter the content of unesterified cholesterol in wild-type human fibroblasts over 2 days (Figure 8). In contrast, U18666A (a prototypic lysosomotropic tertiary amine) promoted massive sterol accumulation, as has been widely reported [36]–[38].

**Figure 9:**
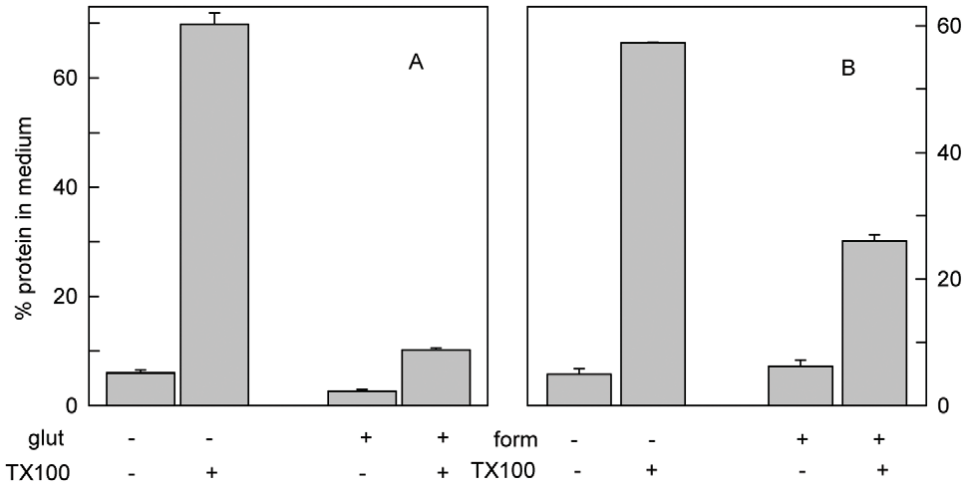
Release of proteins from cells fixed by aldehydes. NPC1 cells were grown to confluence in 6-well plates, the monolayers rinsed and then incubated for 1 h on ice with 0.4 ml PBS with or without 1% glutaraldehyde (glut, panel A) or 4% formaldehyde (form, panel B). The wells were then rinsed and incubated for 20 min at 37°C with PBS with or without 0.1% TX-100. The contents of input wells were harvested in 0.1% in Triton X-100 plus 0.1 N. NaOH. To measure protein release in the other wells, the overlying medium in wells incubated with or without 0.1% Triton X-100 was collected and saved, and the adherent residues harvested in 0.1 N. NaOH. Before assay, all samples were adjusted with concentrates to 0.1% Triton X-100 and 0.1 N. NaOH, then treated with 5% trichloroacetic acid and spun. The precipitates were washed once with 5% trichloroacetic acid, dissolved in 0.1 N. NaOH and the protein assayed. Summing the media and pellets indicated full recovery of the input protein. Values are averages of duplicate determinations on duplicate wells ± average deviation in a representative experiment.

**Figure 10:**
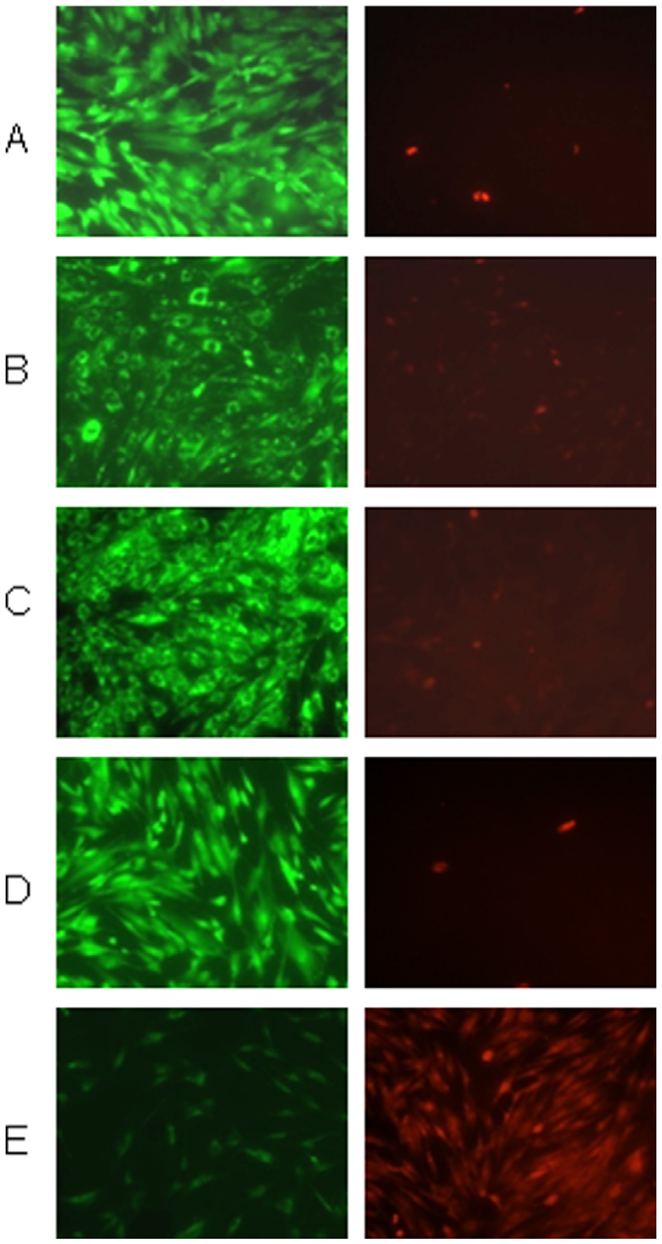
Permeability of fixed NPC1 cells: Live/Dead assay. Cells of NPC1 line 93.59 were grown on glass cover slips, rinsed and treated as described below. They were then incubated with 3 µM calcein AM plus 5 µM ethidium D homodimer for 30 min at room temperature, rinsed and inverted onto glass slides for fluorescence microscopy using a Zeiss Axiovert 100 microscope. Left column, calcein fluorescence; right column, ethidium fluorescence. Row A: untreated fibroblasts. Row B: Cells were incubated with 3.2% formaldehyde in PBS for 30 min on ice, quenched with 50 mM NH_4_Cl for 10 min at room temperature and rinsed. Row C: Cells were incubated with1% glutaraldehyde in PBS for 30 min on ice, quenched with 20 mM glycine in PBS for 10 min at room temperature and rinsed. Row D: Cells were incubated in growth medium containing 20 µM CPZ for 1 h, then rinsed and stained. Row E: Cells were incubated with glutaraldehyde as in panel C, then washed and incubated with 0.1% Triton X-100 in PBS for 15 min at room temperature, rinsed and stained.

We also considered the possibility that fixation with glutaraldehyde and formaldehyde mobilized membrane cholesterol by disrupting or permeabilizing the cells. However, we observed that fixed cells retained their morphological integrity (Figure 6). Furthermore, fixation did not increase the shedding of cytoplasmic proteins but rather prevented their release, even from cells lysed with Triton X-100 (Figure 9). Finally, Live/Dead analyses showed that only a small fraction of NPC1 cells that were fixed with either glutaraldehyde or formaldehyde allowed calcein to exit or ethidium to enter the cell interior (Figure 10).

**Figure 11:**
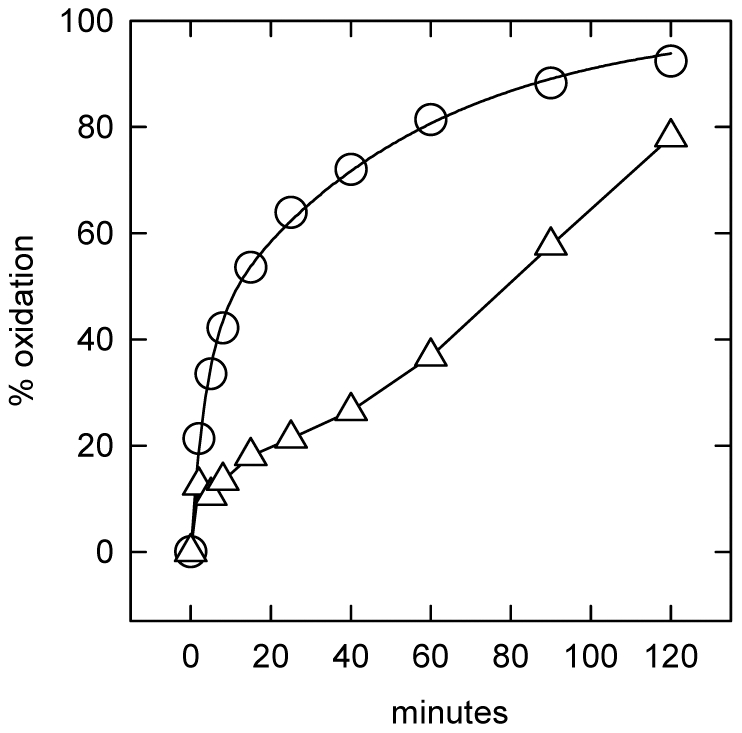
Effect of lysophosphatidylserine on cholesterol dynamics in fixed NPC1 fibroblasts. Cells of NPC line 93.59 were suspended, fixed, washed, resuspended to 1.2 mg protein in 0.5 ml PBS (pH 6.6) and incubated for 5 min at 37°C without (○) or with (Δ) lysophosphatidylserine (150 µM; ∼0.8 mol/mol cholesterol). The time course of cholesterol oxidase action was then analyzed as described for Method B in Materials and Methods. The half-times for the fast and slow steps in the cells lacking lysophosphatidylserine were estimated to be 2.5 and 36 minutes, with 39% of the cholesterol in the fast compartment and 61% in the slow compartment. One of two similar experiments is shown.

We also obtained indirect evidence that the mobilization of intracellular cholesterol by fixation in fact reflects its activation. For this, we treated fixed cells with a lysophosphatide because such membrane-intercalating agents associate with and thereby reduce the activity of membrane cholesterol [20], [23]–[25]. As shown in Figure 11, lysophosphatidylserine strongly inhibited the oxidation of cholesterol in glutaraldehyde-fixed NPC1 cells. (Accelerating time courses for the action of cholesterol oxidase on membrane cholesterol such as that seen in Figure 11 have been observed previously [26]. This effect may be related to the stimulation of the reaction by the reaction product, cholest-4-en-3-one (cholestenone), presumably through the displacement of substrate cholesterol from its association with membrane phospholipids and its consequent activation [39].) Note that cholesterol oxidase should not be able to access the LE/L in fixed cells, even if their plasma membranes were leaky; thus, the mechanism of action of the aldehydes is likely to be through cholesterol activation and mobilization.

[A cautionary note: That glutaraldehyde fixation does not inhibit but actually increases the flux of intracellular cholesterol to the cell surface can account for high estimates previously reported for the fraction of cell cholesterol associated with the plasma membrane; namely, up to 94% in fixed wild-type fibroblasts [40]. That is, the fixation used in those analyses presumably promoted the circulation of intracellular cholesterol to the cell surface during prolonged incubations with cholesterol oxidase. Thus, the high mobility of intracellular cholesterol, even in fixed cells, can complicate the analysis of its *in situ* distribution and dynamics. Unfortunately we know of no general inhibitor of intracellular cholesterol movement other than cold temperature.]

## Discussion

The transport of cholesterol molecules among cellular membrane compartments, including the LE/L, normally proceeds with time constants on the order of tens of minutes or less, apparently mediated by a variety of transfer proteins [7], [20], [41]–[43]. Despite the absence of a functional NPC1/NPC2 pathway, the large cholesterol pool in the LE/L of NPC fibroblasts is also mobile, reaching probes at the cell surface with an apparent half-time of a few hours; see Results and [10], [11]. Other evidence for the mobility of LE/L cholesterol in NPC1 cells is that these compartments can be emptied simply by growing the cells without serum lipoproteins for a few days; see, for example, [44].

We now report that CPZ and 1-octanol increased the mobility of LE/L cholesterol in both NPC1 and NPC2 fibroblasts. The actions of these amphipaths were uniformly strong in one cell line (NPC1 93.59), moderate in another (NPC1 93.41) and variable in a third (NPC2 99.04); these differences are consistent with the significant variations seen in the phenotypes of NPC individuals [45], [46]. It seems likely that amphipaths mobilize NPC LE/L cholesterol by displacing it from strong associations with the bilayer lipids, thereby enhancing its chemical activity (see Introduction). This mechanism can explain why sphingomyelin, which binds cholesterol avidly, inhibits the ability of NPC2 to catalyze the removal of the sterol from bilayers while ceramides, which displace cholesterol competitively from its association with phospholipids [25], stimulate this process [47]. The ability of 25-hydroxycholesterol to increase the egress of cholesterol from the LE/L of NPC1 cells [11], [48] presumably also reflects amphipath-mediated activation [49], [50], given that this oxysterol also promotes the reactivity of cholesterol with cholesterol oxidase as well as its transfer to β-cyclodextrin and to the endoplasmic reticulum [24], [51], [52].

Membrane-intercalating amphipaths that affect the disposition of cell cholesterol can be grouped in three classes. The term class 1 amphipaths has been used to designate agents that mimic the effect of excess cell cholesterol [53]; it appears that this effect often reflects their ability to displace membrane cholesterol from phospholipid complexes and thereby to activate it. Class 1 includes octanol, CPZ, 25-hydroxycholesterol and dozens of other amphipaths [19], [22], [25], [49], [50]. Class 2 amphipaths such as U18666A appear to sequester sterol in the LE/L and thereby reduce plasma membrane cholesterol [11], [36]–[38], [53]. A third group of intercalators, which we shall call class 3 amphipaths, reduce the activity of bilayer cholesterol, apparently by forming complexes with it. Class 3 includes phospholipids, lysophosphatides and hexadecylphosphocholine [19], [20], [26], [54].

CPZ has sometimes been considered to be a class 2 agent because of its potential to induce LE/L lamellar body formation or “lipidosis” [33], [34]. However, it does not fit well into this category [34]. In particular, CPZ does not cause cholesterol accumulation in wild-type human fibroblasts as do typical class 2 agents (Figure 8). Rather, it behaves as a class I (cholesterol-activating) amphipath; see Results and [25]. The ability of CPZ to stimulate SCAP may also reflect this ability [55], [56]. In contrast, the prototypic class 2 agent, U18666A, stimulates the accumulation of LE/L cholesterol (e.g., Figure 8 and [11], [36]–[38], [53]) but does not increase cholesterol esterification [57], promote SCAP activation [56] or activate red cell or fibroblast cholesterol (not shown). We therefore infer that CPZ is a strong class I agent and, at best, a weak class 2 agent, while the reverse is the case for U18666A.

To our surprise, glutaraldehyde and formaldehyde did not block the accessibility of intracellular cholesterol to cyclodextrin (Figure 3) or cholesterol oxidase (Figure 4) but, in fact, stimulated it. Since aldehydes do not chemically modify sterols or bilayer lipids other than aminophospholipids (see p. 33 in [58]), their action here would seem to be indirect.

We suggest that the mechanism involves activation of LE/L cholesterol [26], [27], [28]. This premise is supported by the reversal of the glutaraldehyde effect by the cholesterol-complexing intercalator, lysophosphatidylserine (Figure 11). Furthermore, we found that the transfer to HPCD of both plasma membrane and intracellular [^3^H]cholesterol in fixed NPC cells was associated with a high energy of activation, ∼25 Kcal/mole (not shown). These high activation energies are comparable to those observed with unfixed cell membranes and bilayer vesicles [59], suggesting that glutaraldehyde-stimulated efflux faces the same hydrophobic barrier (namely, cholesterol hydration) as obtains in normal bilayers.

How cholesterol traverses the fixed cytoplasm in an accelerated fashion is mysterious, given that cytosolic shuttle proteins should be immobilized by glutaraldehyde. However, even in fixed cells, cholesterol could diffuse along bilayers to the protein-based junctions that bridge apposed organelle membranes; downhill transfer could then occur at these sites [7], [41], [43], [60], [61]. Alternatively, cytoplasm fixed by glutaraldehyde is permeable to small molecules; see, for example, the experiment with calcein AM shown in Fig. 10C; also ref. [62]. It is therefore conceivable that sterols are transported by small aqueous shuttles; however, evidence for such carriers is lacking. A case can be made for the unmediated desorption of sterol molecules from bilayers followed by their aqueous diffusion to acceptors [63], [64]. But the characteristic time for such transfers is usually a few hours; in one study, the unaided exit of cholesterol from washed lysosomes took days [65]. Still, the desorption of active membrane cholesterol might be more rapid. Finally, we note that the collisional transfer of membrane cholesterol, at least to β-cyclodextrin, can proceed with a t_½_ of seconds or less [59] and is stimulated both by amphipaths and by glutaraldehyde, presumably through its activation [11], [20], [23]–[25]. Thus, collisional transfers between closely-spaced donors and acceptors in fixed cells might still occur on a time-scale of several minutes.

In conclusion, we have demonstrated that, in fibroblasts lacking functional NPC1 or NPC2 proteins, cholesterol exits the surface and lumenal membranes of the LE/L by an alternate route. This pathway (or pathways) is stimulated when cholesterol is activated both by cross-linking with aldehydes and by membrane-permeable class 1 amphipaths. While glutaraldehyde may not be a facile reagent for the study of intracellular cholesterol transport, the effects reported here nevertheless constrain our thinking about this process. In addition, our findings suggest that drugs that activate LE/L cholesterol stores might offer a useful adjunct in treating NPC disease. In particular, exogenous acid sphingomyelinase releases LE/L cholesterol from its high-affinity binding partner [18], [47] and presumably activates it thereby, as is seen with other sphingomyelinases [66], [67]. Acid sphingomyelinase therapy might therefore be potentiated by well-chosen sterol-activating (class 1) amphipaths. The mobilization of sequestered LE/L cholesterol by β-cyclodextrins [8], [9], [13]–[16] might similarly be enhanced by class 1 amphipaths, given that these agents stimulate the net transfer of intracellular cholesterol to HPCD; see Figure 5 and [23], [24]. Scores of amphipaths of different types activate membrane cholesterol [25]. Thus, selected drugs like CPZ (Thorazine®) that cross the blood-brain barrier and, being lysosomotropic, accumulate in acidic LE/L compartments might prove to have value in the treatment of NPC disease.

## Materials and Methods

### Materials

Cholesterol, 1-octanol, CPZ, cholesterol oxidase (*Streptomyces* sp), filipin III, HPCD (ave. MW = 1396) and glutaraldehyde were purchased from Sigma-Aldrich. 1α,2α-[^3^H]cholesterol was from American Radiolabeled Chemicals or Perkin Elmer. U18666A was obtained from Biomol and lysophosphatidylserine from Avanti. The Live/Dead assay kit was from Invitrogen.

### Cell culture and treatments

Normal human foreskin fibroblasts were cultured to ∼90% confluence in DME10; 75 cm^2^ flasks contained ∼2 mg protein and ∼70 µg cholesterol at ∼35±1 µg (SEM, n = 24) cholesterol/mg protein. The two lines of heterozygous human skin fibroblasts lacking detectable NPC1 protein were obtained from Peter Pentchev. The mutations in the heterozygous NPC1 line 93.59 are 275A>G and 395delC (Walter D. Park, personal communication); these alterations create missense and frame shift errors that lead to a severe biochemical phenotype [45], [46]. Our cultures of line 93.59 had 86±4 (SEM, n = 16) µg cholesterol/mg protein. The mutations in the NPC1 line 93.41 are 2926T>C and 3265G>A (Walter D. Park, personal communication); these create missense and splicing errors with a severe biochemical phenotype [45], [46]. Our cultures of line 93.41 had 112±7 (SEM, n = 13) µg cholesterol/mg protein. The NPC2 cells were NIH line 99.04; these cells are homozygous for a G58T substitution that produces an E20X stop codon that blocks NPC2 protein expression and creates a severe biochemical phenotype [46]. These NPC2 cells had 82±2 (SEM, n = 10) µg cholesterol/mg protein. All cells were cultivated in DME10 and were mechanically dissociated following a 1 min incubation at 37°C in PBS (pH 8.0) containing 0.05% trypsin and 0.02% EDTA, as described [68]. The cells were washed and, unless stated otherwise, kept on ice in PBS (pH 7.4). CPZ was introduced in PBS (pH 7.4), while non-polar compounds were in ethanol (≤1% final).

### Magnitude and dynamics of intracellular cholesterol determined by [^3^H]cholesterol exchange (Method A)

As described previously [11], cells in 75 cm^2^ flasks were pulse-labeled with ∼1 µCi of [^3^H]cholesterol and allowed to equilibrate over 1 day of culture (>6 half-times of exchange between cell surface and intracellular compartments). The cells were rinsed, dissociated and re-plated in DME10 in 25 cm^2^ flasks; this step eliminated contamination by stray [^3^H]cholesterol stuck to the labeled growth flasks. After re-attachment for 3 h, the monolayers were rinsed, incubated in PBS (pH 8.0) with or without 1% glutaraldehyde for 20–30 min on ice, rinsed and preincubated with 1 ml PBS (pH 7.4) with or without amphipaths for 5 min at 37°C. This incubation medium was collected for the zero-time point. The flasks were then incubated at 37°C in 1 ml fresh PBS (pH 7.4) with or without amphipaths containing this exchange partner: 38 µg cholesterol carried in 25–28 mg HPCD. (This cocktail was developed empirically so that the cell sterol mass remained nearly constant during the exchange of the [^3^H]cholesterol.) To obtain data points, the overlying medium was harvested and replenished several times during the incubation; this procedure made the exit of [^3^H]cholesterol quasi-irreversible. The supernatant media were cleared by centrifugation and their radioactivity determined. Residual cell radioactivity, cholesterol mass and protein were determined at the end of the time courses. Protein was analyzed in duplicate by BCA (Pierce kit) using a BSA standard; cholesterol was determined by HPLC [68]. Cell attachment and integrity were monitored by phase contrast microscopy during all incubations.

### Determination of the magnitude and dynamics of the intracellular cholesterol compartment by cholesterol oxidase susceptibility at the cell surface (Method B)

Cells were grown to confluence, dissociated from their flasks, washed and aliquots taken for the determination of cholesterol and protein. The remainder was incubated for 20 min on ice with 1% glutaraldehyde in PBS (pH 8.0), washed, resuspended to ∼1 mg protein/ml PBS (pH 6.6) and amphipaths added. The samples were preincubated at 37°C for ∼5 min and time courses then initiated by the addition of cholesterol oxidase to 40 IU/ml. [A high level of cholesterol oxidase was used to accentuate the difference between the oxidation of the cell surface (fast) and intracellular (slow) pools.] At intervals, aliquots were extracted for the analysis by HPLC of the mass of the remaining cholesterol and the cholest-4-en-3-one (cholestenone) product [68].

### Analysis of kinetic data

The time courses for cell [^3^H]cholesterol exchange (Method A) and for the interaction of cell cholesterol with cholesterol oxidase (Method B) were clearly not first-order but conformed well to the bi-exponential expression, y = ae^−bt^+ce^−dt^ (see, for example, Figs. 2, 3 and 4). Least-squares best fits were obtained after assigning an infinite time point which was 93–102% of transfer or oxidation. Best fits to this equation all had R^2^ values greater than 0.99. We took the coefficients a and c in the expression to represent the sizes of the cell surface and intracellular cholesterol compartments, respectively. The apparent first order rate constants, b and d, yielded values for the half-times of the fast and slow processes respectively; that is, t_½_ = ln 2/b and t_½_ = ln 2/d.

Because the half-times for the reaction of the intracellular pool are complex functions of its movement between the cell surface and intracellular compartments plus the unidirectional exit or oxidation step, true rate constants for transfer to and from the LE/L cannot be extracted from the data; see page 15 in [69]. The actual rate constants for the exit of cholesterol from the LE/L presumably exceeded the values suggested by the observed half-times because the inward flux of plasma membrane cholesterol will replenish the LE/L and thereby slow its clearance from that space.
